# Supplementary material for: Garden-path sentences and the diversity of their (mis)representations
Source: PLoS One. 2023 Jul 18;18(7):e0288817. doi: 10.1371/journal.pone.0288817 (PMC10353815; doi:10.1371/journal.pone.0288817)
Supplement: S1 Table — (PDF) [file pone.0288817.s001.pdf]

# Supporting information

**S1 Table.**
**Model examples of incorrect responses to the question *What did the policeman do?* in Experiments 4 and 5.**

| Correct | Type of mistake | | Example |
|---|---|---|---|
| Yes | | | he searched the van |
| | | | he searched the storekeeper's car |
| | | | he searched the van belonging to the storekeeper |
| | | | he checked the van |
| No | I don't know | | I don't know |
| | | | it wasn't mentioned |
| | | | nothing |
| No | Substitution | GP | he searched the storekeeper |
| | | | he searched the storekeeper and the van |
| | | Other | he searched the policeman |
| | | | he searched the station |
| | | | he searched the student |
| | | | he stole the van |
| No | Different | | in front of a shop |
| No | Other | Incomplete | he searched |
| | | | he searched something |
| | | Other | h |
| | | | jdfak |
