# Supplementary material for: Garden-path sentences and the diversity of their (mis)representations
Source: PLoS One. 2023 Jul 18;18(7):e0288817. doi: 10.1371/journal.pone.0288817 (PMC10353815; doi:10.1371/journal.pone.0288817)
Supplement: Attachment — Submitted filename: Response to reviewers.docx [file pone.0288817.s003.docx]

**Response to reviewers**

REVIEWER #1

The manuscript has considerably improved with regard to a number of issues raised in the previous review round. In particular, the authors have included a justification for replicating the two experiments based on whole-sentence presentation (Experiments 2 and 4) with self-paced reading (Experiments 3 and 5). The revised version also includes a clearer discussion of how the findings relate to theoretical accounts of lingering interpretations. Finally, the authors have also made a number of useful revisions to the background section which considerably improve readability and clarity of the manuscript.

*** We thank the reviewer for their positive feedback, especially for the note about the improved readability and clarity of the manuscript.

However, a crucial issue which definitely has to be addressed before publication of the manuscript can be considered is how data trimming/outlier exclusion was conducted in the statistical analyses, particularly for the critical regions in the two self-paced reading experiments. The analyses currently reported in the manuscript are based on an extremely liberal criterion for outlier exclusion, with extremely long reading times which clearly do not reflect natural reading still included in the analysis. A brief reanalysis for the reading time data from Experiment 3 that I have conducted with the data set provided on OSF suggests that the results may indeed be influenced by a small number of extreme values which have survived data trimming. I elaborate on this issue in considerable detail below, and have included some suggestions for how this issue can be fixed. Another, relatively minor issue which should still be addressed is that, while readability of the manuscript overall has considerably improved, the Results sections for the five experiments are still difficult to follow and contain a multitude of tables and figures.

*** We are grateful to Reviewer 1 for these comments. We changed the trimming of data in both the word-by-word SPR experiments in the revised version of the manuscript (see below in detail).

In sum, I have read the revised version with great interest, and I believe that the five experiments reported in the manuscript potentially constitute a valuable contribution to the field. That said, the issue of outlier exclusion strikes me as particularly important: It obviously makes a crucial difference whether the garden-path effects reported in the manuscript really exist, or whether they are caused by a somewhat larger proportion of extreme values in the garden-path than in the control conditions. The issue therefore has to be addressed in a responsible manner.

*** Thank you. The more conservative trimming procedure which we used in the revised version of the paper yielded exactly the same pattern of results as the previous liberal trimming (see below in detail).

I. **Outlier Exclusion**

- In the first version of the manuscript, the authors had relied on an extremely liberal method for outlier exclusion, and had excluded only very extreme reading times above 15000ms. Even in the revised version, the cut-offs applied (6500ms for Experiment 3, 5500ms for Experiment 5) are far too liberal for web-based self-paced reading experiments of this kind. Given that the critical disambiguating regions in these experiments consist of a single 6-8 letter word, even a reading time of more than 2 seconds is a clear indication that the measure does not reflect natural reading. For the present study, however, even a reading time of 5000 ms is not excluded from the analysis. The authors justify their approach to outlier exclusion by arguing that they have followed the recommendations from Baayen & Milin’s (2010) tutorial for reaction time analysis. However, applying the approach suggested in this tutorial to the present data set mechanically (i.e. without considering what the measures actually represent in this specific study) strikes me as not quite suitable, for at least two reasons: First, Baayen & Milin’s recommendation to only conduct minimal data trimming and to remove only clearly discontinuous reading times refers to the analysis of reaction times in general, and does not take into account what these reaction time measures represent in a concrete study (such as properties of the stimulus for which the reaction times were measured). Thus, their recommended approach is based exclusively on the distribution of data points, and does not discuss any content-based common-sense criteria for what constitutes an outlier. For the reading times measured in the present study, we know that a healthy, fully literate, adult native speaker of a language should definitely be able to read a single 7-letter word in considerably less than 2 seconds. As a result, an extreme reading time of, for instance, 5000ms should definitely be considered an outlier, irrespective of the distribution of data points. Second, Baayen & Milin’s tutorial refers to experimental reaction time studies conducted in a controlled lab environment. Data sets from such studies typically contain only a relatively small number of outliers because the controlled surroundings ensure that a participant only rarely gets distracted from the task. In the current web-based study, in contrast, the experimenter had no control of the participant’s surroundings. As a result, outliers are a potentially much more severe issue in web-based studies. In sum, I thus strongly recommend that the authors revise their analyses and rely on an established approach to outlier exclusion used in previous self-paced reading studies (e.g. first exclude extreme values which clearly do not reflect natural reading, then also exclude reading times which are more than 2 standard deviations above or below the overall mean reading time for the respective segment).

Note that the influence of outliers is a particularly crucial issue here, particularly given that linear-mixed effects models are known to be fairly vulnerable when it comes to the influence of a small number of extreme data points. I thus conducted a brief analysis for the critical disambiguating segment in Experiment 3 myself, with the data provided by the authors on OSF. The results from this more conservative analysis suggest that the crucial effect of ambiguity reported for the disambiguating segment in Experiment 3 is at least partly driven by the influence of a small number of outliers in the data, and becomes considerably smaller when these outliers are excluded: If I conduct the analysis in the same way as in the manuscript (i.e. exclusion of data points above 6500 ms and below 150 ms), mean reading times by condition for the disambiguating segment (i.e. Segment 7) obviously show the very same data pattern as in Figure 2:

Garden-path, animate: 694 ms (SD: 642)

Non-garden-path, animate: 602 ms (SD: 462)

Garden-path, inanimate: 625 ms (SD: 521)

Non-garden-path, inanimate: 548 ms (SD: 376)

These results show clear numerical trends for a garden-path effect, with a 92 ms difference between garden-path- and non-garden path trials for animate items and 77 ms for inanimate items (Note, however, that the huge standard deviations already suggest a presence of a small number of extreme data points.). However, if data points which are more than 2 standard deviations above or below the overall mean reading time for the segment are excluded (an established procedure for outlier exclusion used in a number of previous reading studies), the respective garden path-effects are very considerably smaller, with only a 27 ms difference for animate and 25 ms for inanimate items:

Garden-path, animate: 558 ms (SD: 303)

Non-garden-path, animate: 531 ms (SD: 272)

Garden-path, inanimate: 534 ms (SD: 275)

Non-garden-path, inanimate: 509 ms (SD: 234)

In sum, all this calls for a through exploration of the potential influence of outliers in the data, for all experiments reported in the manuscript. It is crucial to determine whether the key effects reported in the manuscript really reflect longer average reading times for garden-path- than for non-garden-path sentences, or whether the effect is instead driven by a slightly higher number of outliers in the garden-path conditions.

*** We would like to thank the reviewer for such a thorough comment. We fully agree that the selected data trimming procedure might affect the measured effects and that it therefore is a delicate issue. Initially, our approach aimed to exclude only unmistakable outliers. However, we acknowledge the reviewer's point that this method may inadvertently include extreme values resulting from external factors like distraction, rather than solely reflecting processing costs.

In light of the reviewer's recommendations, we proceeded with the following revisions: Firstly, we excluded all response times (RTs) below 150 ms. Secondly, to approximate a normal distribution, we performed a log transformation on the RTs. Thirdly, we determined the upper trimming value as the mean of log-transformed RTs plus 2.5 standard deviations. For Experiment 3, this value corresponded to 1486 ms, while for Experiment 4, it was 1600.2 ms. Subsequently, we reran the models and updated the files on OSF. Importantly, the pattern of results remained entirely consistent, with independent effects of sentence type and object animacy once again observed.

Additionally, we have also revised the data trimming procedure for Experiments 2 and 4, where sentence-as-a-whole presentation was employed, so that it now aligns with the 2.5 * SD criterion.

**Additional minor issues:**

**I.**

- In their response letter, the authors say that, based on reviewer suggestions in the previous review round, they have revised Figures 2 and 5 so that they show only segments after the experimental manipulation, and also display untransformed reading times instead of inverse-transformed values. However, these updated graphs are not included in the revised version of the manuscript. I suspect this is just due to some sort of converting error.

*** Thanks for noticing this! It was our mistake since we forgot to change these figures while submitting the revision. This is now corrected.

**II.**

- The analyses are still considerably too detailed; the revised manuscript contains far too many tables and graphs. Some of the tables should either be merged, included in the supplementary materials, moved to OSF, or deleted entirely (with the key results instead reported in the text).

*** We removed the full models from the Supporting information, since these may be easily reproduced from the data available on OSF. Moreover, Tables 2, 5, and 7 (containing acceptability judgment scores and RTs in Experiments 2 and 4) were merged together. And we also merged Tables 10 and 12 (containing error types in response to qwhat in Experiments 4 and 5). This led to a substantial reduction of the number of tables in the manuscript.

**III.**

- As suggested in the previous review round, the authors have added an additional motivation for re-doing Experiments 2 and 4 with self-paced reading (i.e. Experiments 3 and 5) on page 19 (ll. 752-765). While it strikes me as reasonable to motivate these studies with reference to the possibility to look at garden-path effects separately for each segment, arguing that self-paced reading is interesting because it rules out the possibility to go back to a previous segment of the sentence feels a bit weird to me. This essentially characterizes self-paced reading as an artificial research method which does not allow participants to read the sentences naturally.

*** We agree that this was not formulated ideally. We now revised and (based on the suggestion of Reviewer 2 also shortened) this part of the text. The current version is:

“Experiment 3 was a replication of Experiment 2 with one important difference. The stimuli were not presented in a sentence-at-once mode, but using a classic word-by-word presentation. Such presentation provide us with important information about the course of processing, which will be crucial for the interpretation of our results. Measuring RTs on specific regions of the sentence can also give us information about additional difficulty in processing that relates to the changes of animacy of the disambiguating noun.”

**IV.**

- In the paragraph discussing issues of statistical power (particularly the number of items per condition each participant encounters during test session) in the Discussion section on page 39, it strikes me as unnecessary to characterize this as a crucial limitation. My suggestion would be to instead include a brief explanation of why it was not possible or problematic to construct a larger set of materials, for instance to do properties of the language.

*** Thanks for this suggestion. We would prefer to leave the part on task adaptation as it is, but we added this text to the last paragraph of General discussion:

“Moreover, the creation of experimental items was already highly constrained due to grammatical factors. As we mention in the section Structure of Czech and the Current Study, the number of verbs that can be plausibly combined with both animate and inanimate patients is limited in Czech, and thus, we had to use each verb and disambiguating noun twice to get the current number of experimental items. To get even more items and thus raise the statistical power, we would also need to repeat the ambiguous nouns since they come from a feminine declension class which contains a relatively closed set of nouns denoting people (i.e., the růže-paradigm).”

Reviewer #2: The authors have carefully revised the manuscript and addressed my concerns very well. I think that the manuscript has much improved, particularly in the interpretation of the evidence. I only have remaining minor comments and suggestions.

*** We would like to thank Reviewer 2 for their valuable comments and suggestions.

**Minor points**

**I.**

1) The manuscript has become quite lengthy. Of course, it is up to the editors to decide if that is fine, but my advice is to shorten it to improve readability. These are some examples of places that may be shortened:

- Detailed descriptions of previous studies in the Introduction;

- Motivation of design of Experiment 3 (lines 752-765);

- Perhaps some more of the overlapping information in Methods of Experiments 3-5;

- Discussion of methodological aspects in the Discussion section (lines 1421-1497) – these are important but can be expressed with fewer words.

*** We reduced the motivation of design of the Experiment 3 and we partly reduced the Hypotheses section of Experiment 5 (by referring to the Hypotheses of Experiment 3). Also, we removed certain parts of the Introduction. However, we would prefer to leave the General Discussion as it is.

**II.**

2) I would like to clarify my minor point #2 from the previous review round. I meant not only individual differences between readers in whether they are susceptible to garden-path effects but also across-trial differences in whether the reader follows the garden path. In other words, I meant that possibly in some trials, the participants did not follow the garden path at all and parsed the sentence correctly in the first place. Perhaps the authors could consider whether any of the findings could be accounted by this processing pattern, rather than by any patterns of recovery from being garden-pathed. However, I do not insist on incorporating this into the manuscript.

*** We agree with the reviewer that this question is interesting. In part, we touch it in the last paragraph of the General discussion where we mention the task adaptation effects. Nevertheless, we believe that our data at hand do not enable us to discuss these issues in depth and since the paper is already too long (as both reviewers point out), we would rather leave it out of our scope.

**III.**

3) Phrasing of some of the hypotheses has become somewhat vague:

- Hypothesis of Experiment 1 (line 573-574): “there will not be a big difference any significant differences in response accuracy”;

- Hypothesis of Experiment 4 (line 945): “there should be no (or little) difference in response accuracy”.

I feel that it is quite vague to discuss “big differences” or “little differences” (unless we are discussing effect sizes, which is not the case here), so wording in terms of statistical significance would be more rigorous.

*** We agree. In the revised version, it was changed accordingly.

**IV.**

4) I apologize for missing this issue at my first reading but I do not agree with the authors’ description of heuristics with reference to Ferreira & Patson (2007). The authors claim that the heuristic is “the tendency to not inhibit the initial misanalysis of garden-path sentences”. But Ferreira & Parson (2007) use the term ‘heuristics’ to describe the parsing mechanisms per se (for example, late closure heuristic, minimal attachment heuristic, reliance on plausibility, etc.) rather than any mechanisms used to deal with conflicting parses. In other words, the initial misanalysis of garden-path sentences is indeed created based on heuristics but the tendency to not inhibit the initial misanalysis is a separate phenomenon.

*** We believe that this is probably a misunderstanding. We do not claim that heuristics is “the tendency to not inhibit the initial misanalysis of garden-path sentences”. In the last version of the paper, there was “It has been pointed out multiple times that one such example of heuristic processing is the tendency to not inhibit the initial misanalysis of garden-path sentences.” which we think is in accordance what Ferreira & Patson (2007) claim. Nevertheless, to avoid such a confusion and to be more precise, we changed “example of heuristic processing” to “outcome of heuristic processing” in the revised version of the manuscript.

**V.**

5) I suggest some editorial changes:

- Lines 1084-1085: “These results are once 1084 again in line with previously mentioned accounts of garden-path sentence processing” – Please clarify which accounts are meant.

*** We added several citations to this sentence.

- Line 1322-1323: “Slattery and colleagues [21] do not claim that reanalysis of GP sentences does not fail occasionally” – Double negation is difficult to process, please rephrase.

*** Changed to “Slattery and colleagues [21] do not claim that reanalysis of GP sentences is always successful.”

- Lines 1337-1339: “It has already been shown that comprehenders differ tremendously in what (if any) re-reading strategies they use during reanalysis of GP sentences [42,43]” – This sentence looks stranded in this particular spot. I would consider moving this idea to the new paragraph on individual differences (line 1410 and further).

*** Thanks for the suggestion. We moved the sentence to the paragraph about individual differences.

**VI.**

6) Please proofread the paper for punctuation and possibly typos, for example:

- Line 270: “open ended” – A hyphen is missing.

*** Hyphen added.

- Lines 452-454: “as well as the experimental items being created specifically for the purposes of our experiments and did not come from actual usage” – Perhaps “were created” is meant.

*** The paragraph was revised. The current version is:

“As Experiment 1, we ran a pilot study using the acceptability judgment task. Since the structure we worked with has not, to our knowledge, been used in any previous work, and the experimental items were created specifically for the purposes of our experiments, we needed to ensure that the unambiguous versions of the sentences will be considered unproblematic by the readers.”

- Line 1086: “However, we would like to note, that we also detected” – There is an unnecessary comma.

*** Comma removed.

- Lines 1214-1215: “for example while answering the comprehension questions” – A comma is missing.

*** Comma added.

- Lines 1274-1275: “(they successfully attached the 1274 disambiguating noun to the structure and they also reanalyzed the ambiguous noun.)” – The full stop should follow the bracket.

*** Corrected.

- Line 1346-1347: Perhaps there should be no paragraph break here.

*** Agreed. The two paragraphs are now merged.

- Line 1371: “similarity based interference” - A hyphen is missing.

*** Corrected.
